# Autophagic degradation of IRF3 induced by the small-molecule auranofin inhibits its transcriptional and proapoptotic activities

**DOI:** 10.1016/j.jbc.2021.101274

**Published:** 2021-10-05

**Authors:** Anna Glanz, Sukanya Chakravarty, Shumin Fan, Karan Chawla, Gayatri Subramanian, Tia Rahman, Dean Walters, Ritu Chakravarti, Saurabh Chattopadhyay

**Affiliations:** 1 Department of Medical Microbiology and Immunology, University of Toledo College of Medicine and Life Sciences, Toledo, Ohio, USA; 2 Department of Physiology and Pharmacology, University of Toledo College of Medicine and Life Sciences, Toledo, Ohio, USA

**Keywords:** innate immunity, interferon regulatory factor 3, interferon, autophagy, auranofin, apoptosis, inflammatory genes;, fatty liver disease, HBSS, Hank's balanced salt solution, HFD, high-fat diet, HSV-1, herpes simplex virus, IFN, interferon, IRF3, IFN regulatory factor 3, ISG, IFN-stimulated gene, LPS, lipopolysaccharide, NSD, NS-degradasome, PA, palmitic acid, RIPA, RLR-induced IRF3-mediated pathway of apoptosis, VSV, vesicular stomatitis virus

## Abstract

The ubiquitously expressed transcription factor interferon (IFN) regulatory factor 3 (IRF3) is critical for the induction of antiviral genes, *e.g.*, type-I IFN. In addition to its transcriptional function, IRF3 also activates a nontranscriptional, proapoptotic signaling pathway. While the proapoptotic function of IRF3 protects against viral infections, it is also involved in harmful immune responses that trigger hepatocyte cell death and promote liver disease. Thus, we hypothesized that a small-molecule inhibitor of the proapoptotic activity of IRF3 could alleviate fatty-acid-induced hepatocyte cell death. We conducted a high-throughput screen, which identified auranofin as a small-molecule inhibitor of the proapoptotic activity of IRF3. In addition to the nontranscriptional apoptotic pathway, auranofin also inhibited the transcriptional activity of IRF3. Using biochemical and genetic tools in human and mouse cells, we uncovered a novel mechanism of action for auranofin, in which it induces cellular autophagy to degrade IRF3 protein, thereby suppressing IRF3 functions. Autophagy-deficient cells were unable to degrade IRF3 upon auranofin treatment, suggesting that the autophagic degradation of IRF3 is a novel approach to regulate IRF3 activities. Using a physiologically relevant *in vitro* model, we demonstrated that auranofin inhibited fatty-acid-induced apoptotic cell death of hepatocytes. In summary, auranofin is a novel inhibitor of IRF3 functions and may represent a potential therapeutic option in diseases where IRF3 is deleterious.

Interferon (IFN) regulatory factor 3 (IRF3), a key transcription factor, is involved in the synthesis of IFN and the IFN-stimulated genes (ISGs) during virus infection (1). Studies using *Irf3*^*−/−*^ mice have demonstrated that IRF3 is required to protect against a wide range of viruses. IRF3 mediates the innate antiviral response through the rapid induction of type-I IFN, *e.g.*, IFNβ, which protects the infected cells and also establishes the antiviral state in yet uninfected cells (2). In addition to transcriptional function, we have described a direct proapoptotic role for IRF3, the RLR-induced IRF3-mediated pathway of apoptosis (RIPA), which contributes to the overall functions of IRF3 (1, 3, 4, 5, 6). In RIPA, IRF3 is activated by linear polyubiquitination, which triggers its mitochondrial translocation, leading to the activation of the intrinsic apoptotic pathway (5). Using knock-in mice expressing a transcriptionally inactive but RIPA-active Irf3 mutant, we demonstrated that RIPA protects against respiratory viral pathogenesis (5). While the IRF3 functions are protective during virus infection, IRF3 activity can be detrimental to the host in other diseases. For instance, we and others have demonstrated a role for IRF3 in the development of lethal sepsis (7, 8, 9). TLR4 signaling induced by bacterial lipopolysaccharide (LPS) rapidly activates the septic shock response in mice (9). However, the global, as well as myeloid cell-specific, *IRF3*^*−/−*^ mice exhibit increased survival compared with Wt mice. Additionally, IRF3 supports the replication of the parasite *Toxoplasma gondii* by inducing the proparasitic ISGs (10).

While IRF3 clearly causes deleterious effects in sepsis and *T. gondii* infection, its role in liver diseases remains somewhat complex. Early studies demonstrated that ethanol and CCl_4_ trigger ER-stress in hepatocytes to activate a RIPA-like pathway involving the STING-IRF3 signaling axis to exacerbate liver injury (11, 12). In a similar manner, free fatty acids in high-fat diet (HFD)-fed mice activate the STING-IRF3 pathway to cause enhanced inflammation and apoptosis, suggesting that both transcriptional and the proapoptotic activities of IRF3 contribute to the disease pathogenesis (13). In a recent study using nontranscriptional IRF3 expressing mice, we demonstrated that the nontranscriptional activity of IRF3 is sufficient to induce numerous markers of hepatocellular damage in response to ethanol, including increased ALT/AST, triglycerides, and inflammatory cytokine expression (14). In contrast, nontranscriptional IRF3 activity plays a protective role in mice from HFD-induced liver injury partially through the suppression of NF-κB signaling (15).

Given the importance of IRF3 in microbial infection and metabolic disease, it is desirable to identify drugs that can manipulate IRF3 functions to benefit the host. A common strategy to rapidly test many compounds at once is by conducting a high-throughput drug screen. High-throughput screening methods have been used extensively to identify the activators and inhibitors of IRF3 transcriptional pathway (16, 17, 18). However, since both transcription and proapoptotic functions of IRF3 are implicated in viral and metabolic diseases, we sought to conduct a screen that could isolate small-molecule modifiers of IRF3's proapoptotic function. Recently, we performed a high-throughput screen of a library of FDA-approved drugs to identify small-molecule modifiers of RIPA (19). The screen identified doxorubicin and pyrvinium pamoate as two potent agents that promoted the apoptotic function of IRF3. Both doxorubicin and pyrvinium pamoate showed antiviral activities against vesicular stomatitis virus (VSV) and herpes simplex virus (HSV-1) by promoting the apoptotic activity of IRF3. In the current study, we sought to explore the function of a small-molecule inhibitor of the apoptotic activity of IRF3, auranofin, which was identified in our screen. Surprisingly, auranofin inhibited both transcriptional and RIPA functions of IRF3. Finally, we used palmitic acid (PA)-induced cell death in human hepatocytes as a physiologically relevant *in vitro* cell culture model to study the effect of auranofin in a context where both IRF3's transcriptional and nontranscriptional, proapoptotic functions are critical.

## Results

### Auranofin inhibits the proapoptotic and transcriptional activities of IRF3

**Figure 1:**
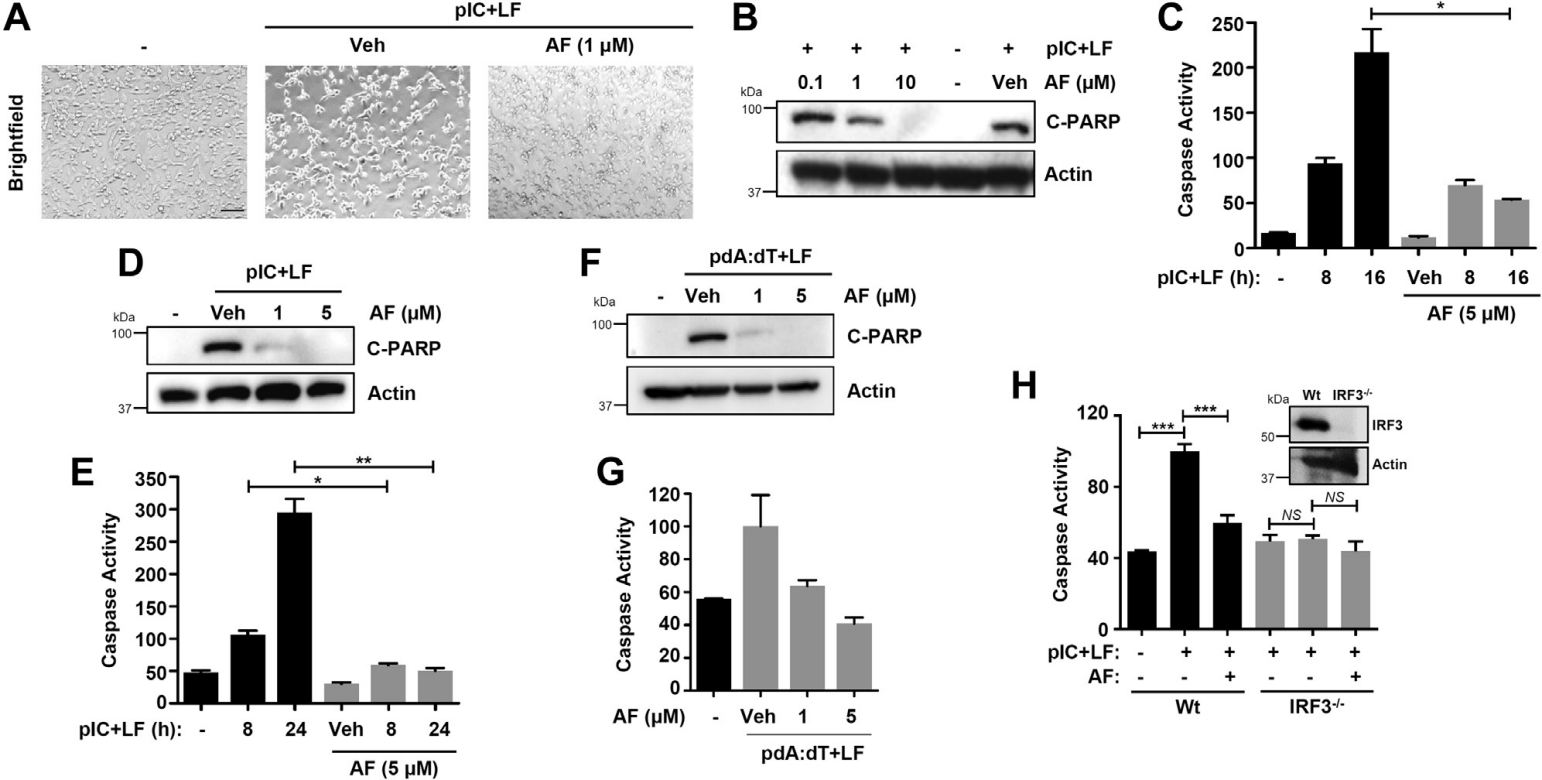
**Auranofin inhibits the proapoptotic activity of IRF3.***A*, HT1080 cells were transfected with polyI:C (pIC+LF) in the absence or the presence of auranofin (AF), and the culture fields were analyzed by bright field microscopy 8 h post-pIC stimulation. *B*, HT1080 cells were transfected with polyI:C (pIC+LF) in the absence or the presence of AF, at the indicated concentrations, for 16 h, when cleaved PARP was analyzed by immunoblot. *C*, HT1080 cells were transfected with polyI:C (pIC+LF) in the absence or the presence of AF for the indicated times when the caspase-3 activity was measured. *D*, MDA-MB-453 cells were transfected with polyI:C (pIC+LF) in the absence or the presence of AF (at the indicated concentrations) for 16 h, when cleaved PARP was analyzed by immunoblot. *E*, MDA-MB-453 cells were transfected with polyI:C (pIC+LF) in the absence or the presence of AF for the indicated times when the caspase-3 activity was measured. *F*, HT1080 cells were transfected with poly(dA:dT) in the absence or the presence of AF for 16 h, when the cleaved PARP was analyzed by immunoblot. *G*, HT1080 cells were transfected with poly(dA:dT) in the absence or the presence of AF for 16 h, when the caspase-3 activity was measured. *H*, Wt and IRF3^*−*/*−*^ HT1080 cells were transfected with polyI:C (pIC+LF) in the absence or the presence of AF for 8 h when the caspase-3 activity was measured. IRF3 protein expression is shown by immunoblot in the inset. Veh, Vehicle (DMSO), ∗ indicates *p* < 0.05, NS, nonsignificant, scale bar, 100 μm.

Recently, we performed a high-throughput screen of a library of FDA-approved drugs to isolate small-molecule activators of RIPA (19). In addition to the activators, our screen identified auranofin as a novel small-molecule inhibitor of RIPA. Auranofin treatment inhibited apoptotic cell death, analyzed by brightfield microscopy, mediated by RIPA in HT1080 cells (Fig. 1*A*). Next, we investigated whether auranofin inhibits the molecular readouts of RIPA. Auranofin strongly inhibited cleaved PARP (C-PARP, Fig. 1*B*) and caspase-3 activity (Fig. 1*C*), dose- and time-dependently, in RIG-I-like receptors (RLR)-stimulated (pIC+LF) HT1080 cells. To inquire whether auranofin inhibits RIPA in other cell types, we used MDA-MB-453, the human cancer cell line, which we previously used to isolate the RIPA-activating compounds. In MDA-MB-453 cells, auranofin significantly inhibited, dose- and time-dependently, the RLR-induced C-PARP (Fig. 1*D*) and caspase-3 activity (Fig. 1*E*). We have previously shown that RIPA can be activated, in addition to dsRNA, by cytoplasmic dsDNA (3). Cytoplasmic dsDNA [poly(dA:dT)]-induced C-PARP (Fig. 1*F*) and caspase-3 activity (Fig. 1*G*) were inhibited by auranofin. Because IRF3 is critical for RIPA, RLR activation in the absence or the presence of auranofin was unable to trigger apoptotic activity in the IRF3 knockout cells (Fig. 1*H*). Therefore, auranofin is a novel inhibitor of the IRF3-mediated apoptotic pathway.

**Figure 2:**
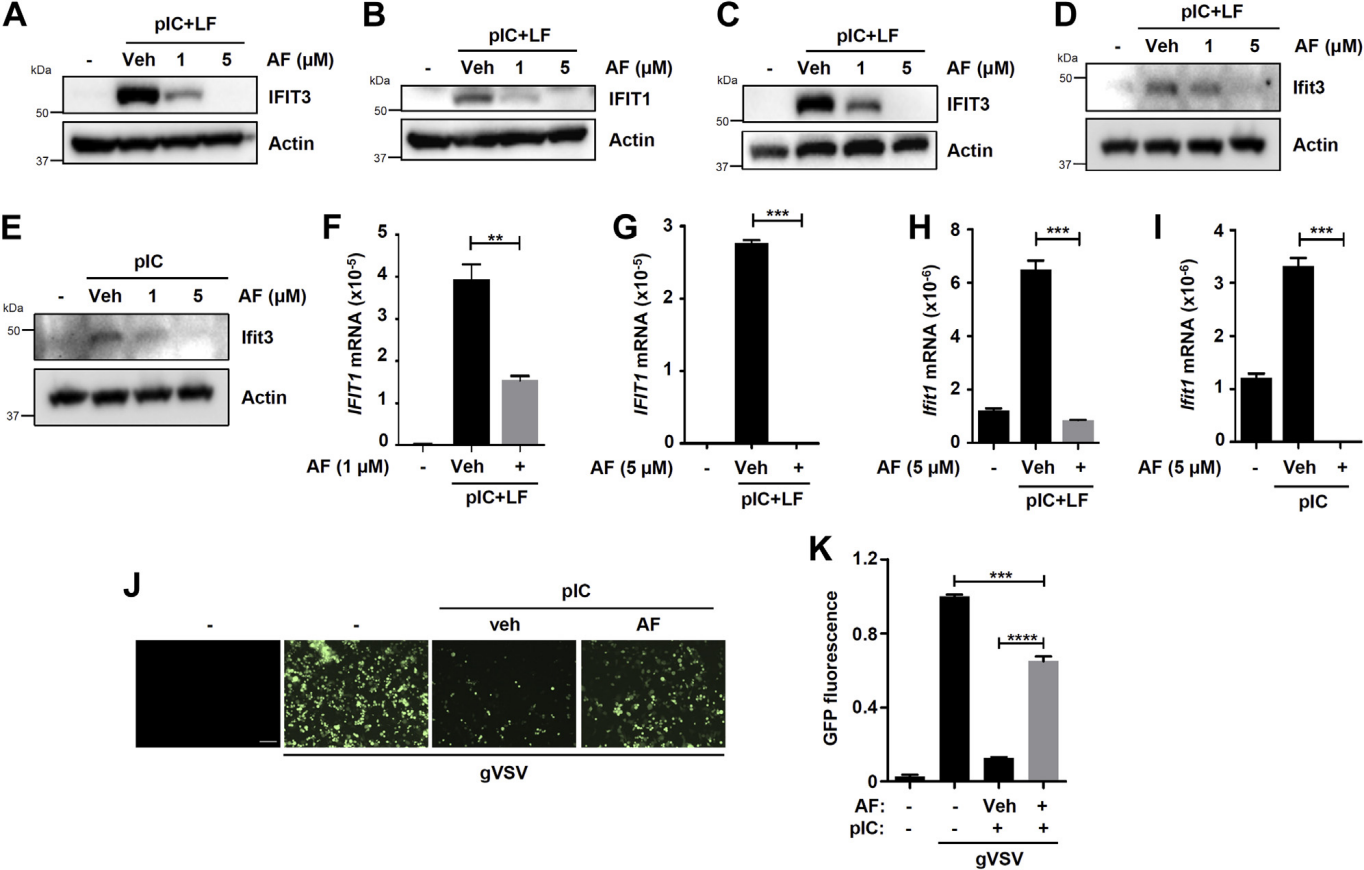
**Auranofin inhibits the transcriptional activity of IRF3.***A–C*, HT1080 (*A* and *B*) or MDA-MB-453 (*C*) cells, pretreated with auranofin (AF), were transfected with polyI:C (pIC+LF) for 16 h, when IFIT3 (*A* and *C*) or IFIT1 (*B*) induction was analyzed by immunoblot. *D* and *E*, RAW264.7 cells, pretreated with AF, were transfected (pIC+LF, *D*) or treated (pIC, E) with polyI:C for 8 h, when Ifit3 induction was analyzed by immunoblot. *F–I*, HT1080 (*F*), MDA-MB-453 (*G*), or RAW264.7 (*H* and *I*) cells, pretreated with AF, were transfected (pIC+LF, *F–H*) or treated (pIC, *I*) with polyI:C for 8 h, when *IFIT1* mRNA induction was analyzed by qRT-PCR. *J* and *K*, HT1080 cells were treated with polyI:C (pIC) in the absence or the presence of AF for 6 h, when the cells were infected with GFP.VSV (gVSV), the viral replication was analyzed by fluorescence microscopy (*J*) or GFP fluorescence of the cell lysates (*K*). Veh, Vehicle (DMSO), ∗ indicates *p* < 0.05, scale bar, 100 μm.

In addition to triggering apoptosis, IRF3 functions as a transcription factor to induce the antiviral genes, such as IFNβ and ISGs (20). We investigated whether auranofin, in addition to RIPA, also inhibits the transcriptional activity of IRF3. In RLR-stimulated cells, the induction of IFIT3 (Fig. 2*A*) and IFIT1 (Fig. 2*B*), the IRF3-dependent genes, was strongly inhibited by auranofin in HT1080 cells. We validated these results in MDA-MB-453 cells, in which auranofin also strongly inhibited the RLR-induced IFIT3 expression (Fig. 2*C*). Similar to the human cells, auranofin also inhibited the RLR-mediated Ifit3 gene induction in the mouse macrophage cell line RAW264.7 (Fig. 2*D*). RLR signaling activates IRF3 in both RIPA and transcriptional pathways, whereas dsRNA-mediated TLR3 signaling activates IRF3 in only the transcriptional pathway, but not RIPA (6, 19). The TLR3-stimulated Ifit3 gene induction was also inhibited by auranofin in RAW264.7 cells (Fig. 2*E*). To inquire whether auranofin inhibits IRF3 activity or triggers the degradation of IFIT proteins, we analyzed the mRNA induction of the IRF3 target genes. RLR-induced IFIT1 mRNA was inhibited by auranofin in HT1080 (Fig. 2*F*), MDA-MB-453 (Fig. 2*G*), and RAW264.7 (Fig. 2*H*) cells. Similarly, TLR3-mediated Ifit1 induction was also inhibited by auranofin in RAW264.7 cells (Fig. 2*I*). Since auranofin inhibited TLR3 and RLR-induced antiviral gene expression, we tested whether auranofin inhibits the antiviral activity of TLR3. PolyI:C treatment (TLR3) inhibited VSV replication, analyzed by both microscopy and GFP fluorescence, and auranofin treatment alleviated the antiviral activity of TLR3 (Fig. 2, *J* and *K*). In summary, auranofin inhibits the transcriptional activity of IRF3, mediated by TLR and RLR signaling pathways, in both human and mouse cells. Together, auranofin inhibits both the proapoptotic and transcriptional activities of IRF3 in multiple cell types.

### Auranofin triggers the degradation of IRF3 protein

**Figure 3:**
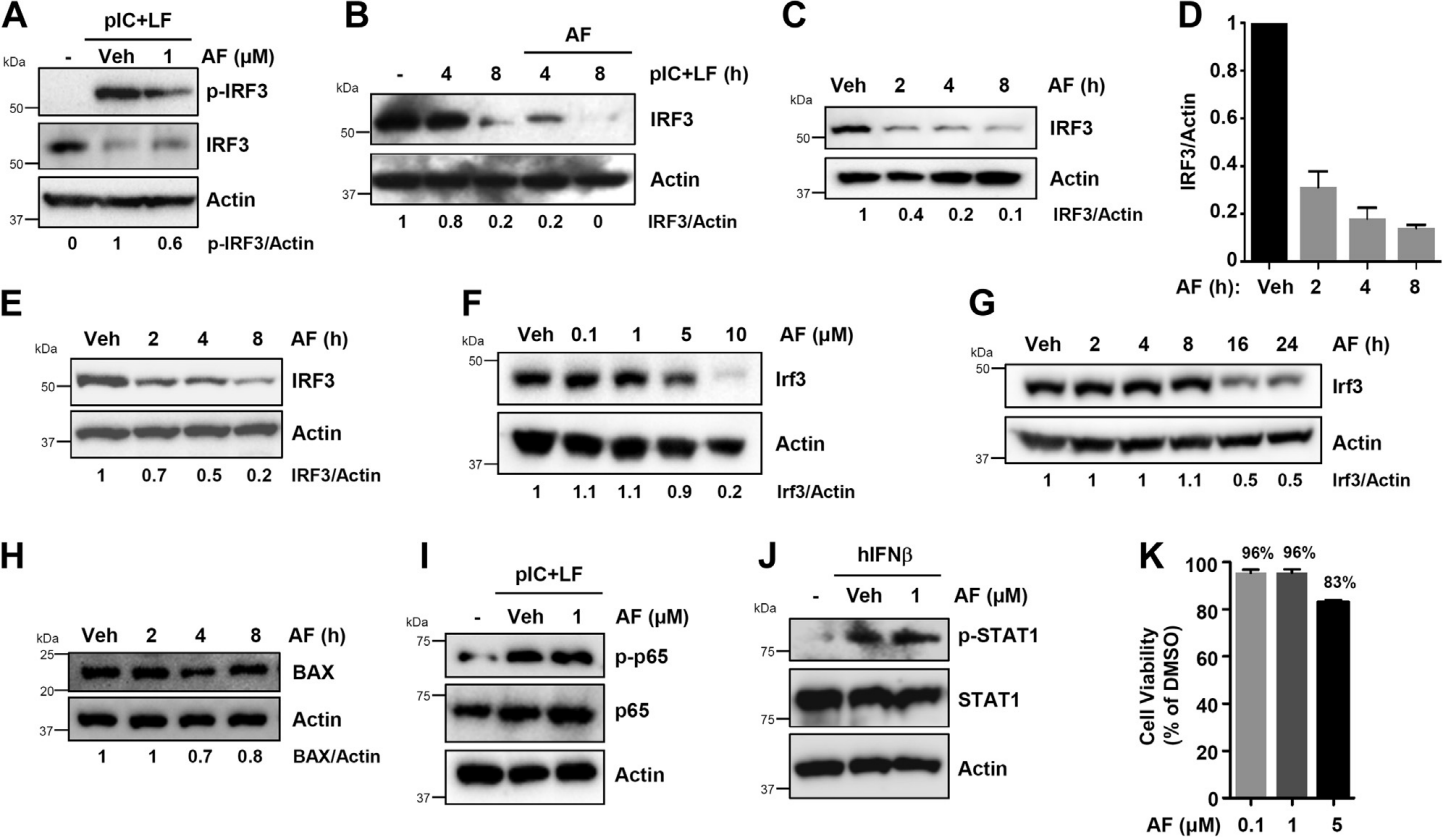
**Auranofin promotes the degradation of IRF3 protein.***A*, HT1080 cells, pretreated with auranofin (AF), were transfected with poly(I:C) (pIC+LF) and the phosphorylated (Ser^396^) and total IRF3 were analyzed by immunoblot after 8 h. *B*, MDA-MB-453 cells, pretreated with AF, were transfected with polyI:C (pIC+LF) for the indicated times when the IRF3 levels were analyzed by immunoblot. *C* and *D*, MDA-MB-453 cells were treated with AF for the indicated times when the IRF3 levels were analyzed by immunoblot (*C*), and the immunoblots from the biological replicates were quantified using ImageJ (*D*). *E*, HT1080 cells were treated with AF for the indicated times when the IRF3 protein levels were analyzed by immunoblot. *F*, RAW264.7 cells were treated with AF at the indicated concentrations for 16 h, when the levels of Irf3 were analyzed by immunoblot. *G*, RAW264.7 cells were treated with AF (5 μM) for the indicated times when the levels of Irf3 were analyzed by immunoblot. *H*, HT1080 cells were treated with AF for the indicated times, and the BAX protein levels were analyzed by immunoblot. *I*, HT1080 cells were transfected with polyI:C (pIC+LF) in the absence or the presence of AF and analyzed for phosphorylated (on Ser^536^) and total p65 by immunoblot after 4 h. *J*, HT1080 cells were treated with interferon (hIFNβ, 1000 U/ml) in the absence or the presence of AF and analyzed for phosphorylated (on Ser^727^) and total STAT1 by immunoblot after 2 h. *K*, HT1080 cells were treated with AF at the indicated concentrations for 8 h when cell viability was assessed by trypan blue exclusion assay. Veh, Vehicle (DMSO).

To determine the molecular mechanism of auranofin-mediated inhibition of IRF3 activities, we investigated the effect of auranofin on IRF3 phosphorylation (p-IRF3), which is critical for the transcriptional activity of IRF3. RLR-induced p-IRF3 (Ser^396^) was inhibited by auranofin (Fig. 3*A*), indicating that auranofin interferes with IRF3 activation. RLR stimulation is known to trigger the proteasomal degradation of IRF3 (21). In MDA-MB-453 cells, RLR stimulation caused robust degradation of activated IRF3, and the auranofin treatment promoted the RLR-induced degradation of IRF3 protein (Fig. 3*B*). These results led to the hypothesis that auranofin promotes the degradation of IRF3 protein, thereby causing the inhibition of its activities. To address this, we tested whether auranofin promotes IRF3 degradation in the absence of RLR stimulation. Our results indicate that auranofin treatment caused degradation of inactive IRF3 protein in a time-dependent manner in different cell types (Fig. 3, *C*–*E*). To validate the auranofin-induced degradation of IRF3 protein, we chose RAW264.7 mouse macrophages, in which auranofin treatment also caused degradation of IRF3 protein in both dose and time-dependent manner (Fig. 3, *F* and *G*). Importantly, auranofin-mediated degradation of IRF3 protein was not dependent on RLR stimulation. BAX, which does not undergo RLR-mediated protein degradation, was largely unaltered in auranofin-treated cells, at least until 8 h posttreatment (Fig. 3*H*). To determine the selectivity of auranofin action, we assessed the auranofin-mediated regulation of other signaling proteins. NF-κB activation, analyzed by phosphorylated p65 (p-p65), remained unaffected by auranofin (Fig. 3*I*). In addition, phosphorylation of STAT1 (p-STAT1), upon IFN-treatment, was unaltered by auranofin (Fig. 3*J*). The effect of auranofin on IRF3 protein was presumably not due to cytotoxicity; the cells maintained >80% viability at the concentrations used throughout the study (Fig. 3*K*). Overall, our results suggest that auranofin treatment can cause the degradation of IRF3 protein in human and mouse cells.

### Auranofin activates the cellular autophagy pathway to induce IRF3 degradation

**Figure 4:**
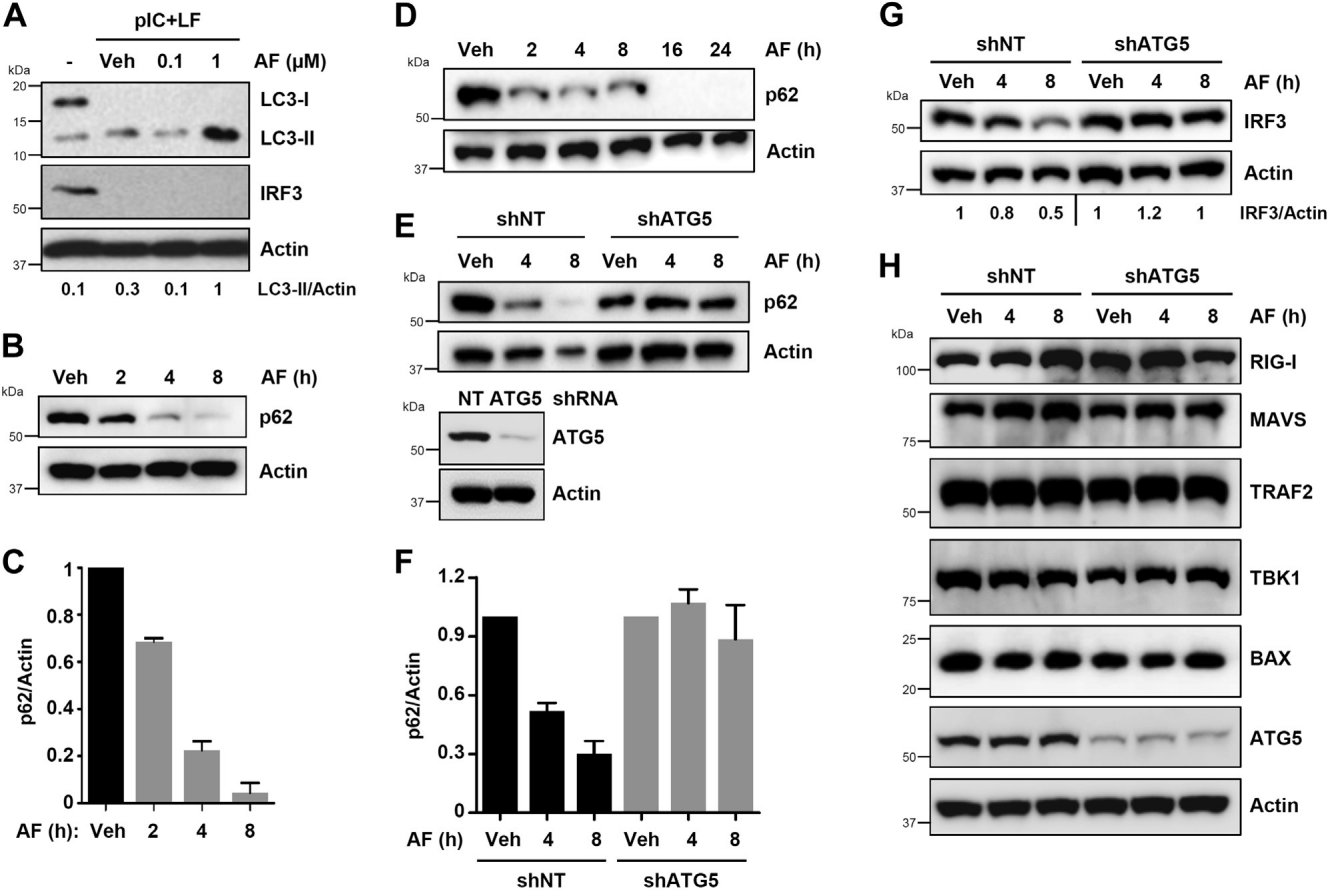
**Auranofin activates the cellular autophagy pathway to degrade IRF3 protein.***A*, HT1080 cells, pretreated with auranofin (AF), were transfected with polyI:C (pIC+LF) for 24 h, when LC3 and IRF3 were analyzed by immunoblot. *B* and *C*, MDA-MB-453 cells were treated with AF for the indicated times when the p62 levels were analyzed by immunoblot (*B*), and the immunoblots from the biological replicates were quantified using ImageJ (*C*). *D*, RAW264.7 cells were treated with AF for the indicated times when the p62 levels were analyzed by immunoblot. *E–G*, HT1080 cells, stably expressing ATG5-specific or nontargeting (NT) shRNA, were treated with AF for the indicated times, when the levels of p62 (*E* and *F*) or IRF3 (*G*) were analyzed by immunoblot. The quantification in *F* is from the biological replicates. ATG5 knockdown levels were analyzed by immunoblot (*E*, *lower panel*). *H*, HT1080 cells, stably expressing ATG5-specific or nontargeting (NT) shRNA, were treated with AF for the indicated times, when the levels of RIG-I, MAVS, TRAF2, TBK1, BAX, and ATG5 were analyzed by immunoblot. Veh, Vehicle (DMSO).

Degradation of IRF3 protein is a key biochemical event regulating virus replication and host inflammatory response. Many viruses also trigger proteasomal degradation of IRF3 to facilitate viral replication (22, 23). We have shown that caspase-mediated proteolysis of IRF3 facilitates its degradation in virus-infected cells (21). In addition, recent studies indicate that the cellular autophagy pathway can suppress the innate immune response by degrading the key signaling proteins, including IRF3 (24, 25, 26). Therefore, we examined whether auranofin-mediated IRF3 degradation depends on the cellular autophagy pathway. We tested whether auranofin, in unstimulated or RLR-stimulated cells, activates autophagy, by analyzing LC3-II accumulation and p62 degradation, the molecular markers of cellular autophagy, which we have used previously (27). Indeed, auranofin treatment caused increased accumulation of LC3-II, a marker of cellular autophagy, in RLR-stimulated cells (Fig. 4*A*). As expected, IRF3 protein was degraded in these conditions (Fig. 4*A*). Next, we tested whether auranofin activated autophagy in the absence of RLR stimulation. Our results indicate that auranofin caused robust degradation of p62, an adaptor protein required for autophagosome-lysosome fusion, in a time-dependent manner (Fig. 4, *B* and *C*). Similar to the human cells, auranofin also triggered time-dependent degradation of p62 in RAW264.7 mouse macrophages (Fig. 4*D*). These results suggest that auranofin activates the cellular autophagy pathway. To validate genetically that auranofin-induced p62 degradation is autophagy-dependent, we used ATG5-knockdown (shATG5) cells (Fig. 4*E*, lower panel), which cannot proceed to the elongation step of the autophagy machinery (27). Auranofin caused robust degradation of p62 in control HT1080 cells, expressing nontargeting (NT) shRNA (Fig. 4, *E* and *F*). However, auranofin-induced p62-degradation was inhibited in ATG5-knockdown cells (Fig. 4, *E* and *F*). Finally, we examined whether auranofin-mediated IRF3 degradation was autophagy-dependent; genetic inhibition of autophagy in the shATG5 cells also protected IRF3 from auranofin-induced degradation (Fig. 4*G*). To examine the selectivity of auranofin-autophagy pathway, we evaluated additional proteins in the RIG-I signaling. The auranofin-autophagy degradation pathway was largely ineffective for RIG-I, MAVS, TRAF2, TBK1, BAX, and ATG5 proteins (Fig. 4*H*). Together, our results indicate that the cellular autophagy pathway is involved in the auranofin-mediated degradation of IRF3 protein.

### Autophagic degradation of IRF3 inhibits its transcriptional and proapoptotic activities

**Figure 5:**
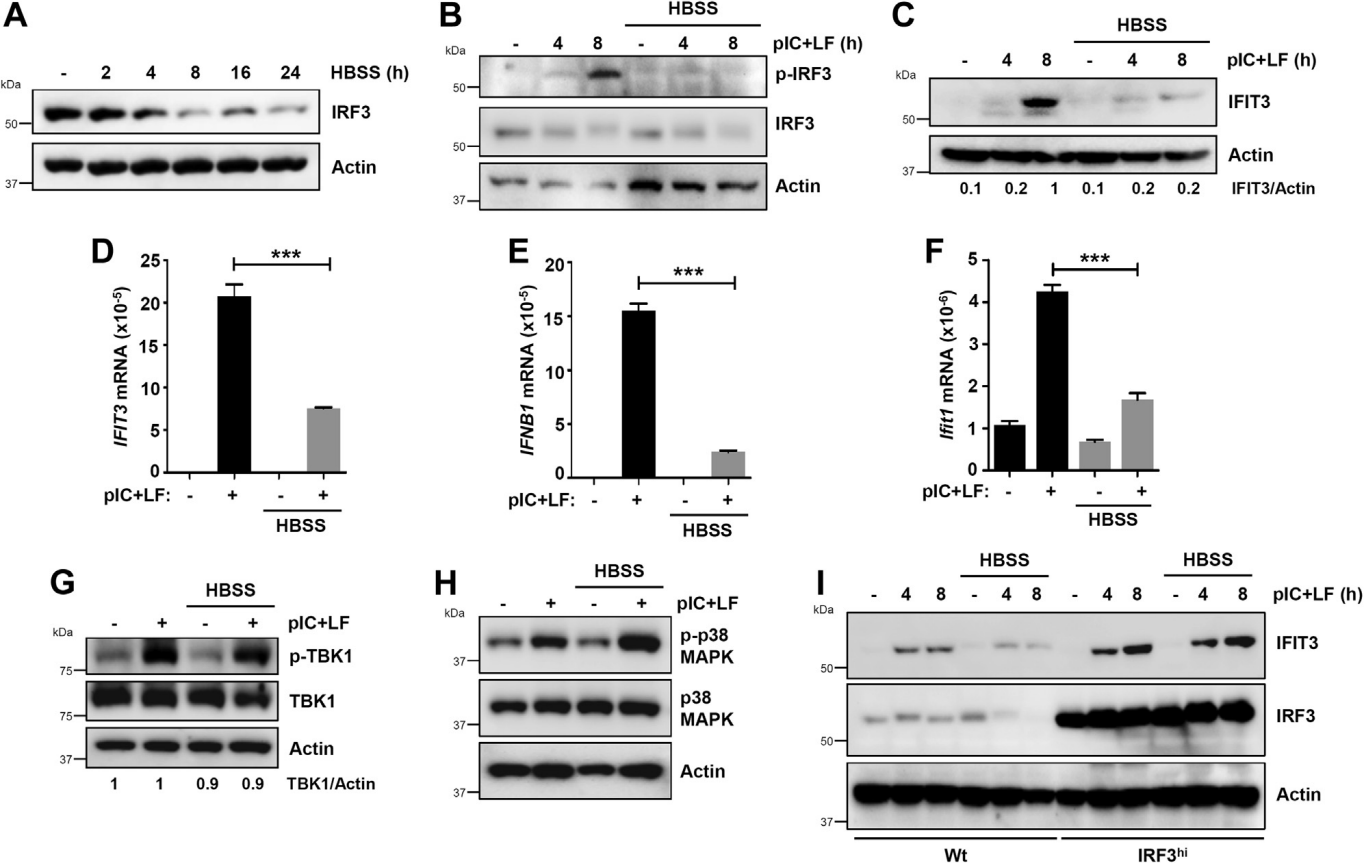
**Autophagic degradation inhibits the transcriptional activity of IRF3.***A*, MDA-MB-453 cells were treated with Hank's balanced salt solution (HBSS) for the indicated times when the protein levels of IRF3 were analyzed by immunoblot. *B* and *C*, MDA-MB-453 cells were transfected with polyI:C (pIC+LF) in the absence or the presence of HBSS for the indicated times, when the protein levels of pIRF3 and IRF3 (*B*) and IFIT3 (*C*) were analyzed by immunoblot. *D–F*, MDA-MB-453 (*D*, *E*) or RAW264.7 (*F*) cells were transfected with polyI:C (pIC+LF) in the absence or the presence of HBSS for 8 h, when *IFIT3* (*D*), *IFNB1* (*E*), and *Ifit1* (*F*) mRNA levels were analyzed by qRT-PCR. *G*, HT1080 cells were transfected with polyI:C (pIC+LF) in the absence or the presence of HBSS and phosphorylated (on Ser^172^), and total TBK1 was analyzed by immunoblot after 4 h. *H*, HT1080 cells were transfected with polyI:C (pIC+LF) in the absence or the presence of HBSS and phosphorylated (on Thr^180^/Tyr^182^) and total p38 MAPK was analyzed by immunoblot after 4 h. *I*, U4C (Wt) and IRF3-overexpressing U4C (IRF3^hi^) cells were transfected with polyI:C (pIC+LF) in the absence or the presence of HBSS, and IFIT3 and IRF3 were analyzed by immunoblot at the indicated times posttransfection. ∗ indicates *p* < 0.05.

In the next series of experiments, we tested whether autophagy-mediated degradation of IRF3 protein inhibits its activities. Nutrient deprivation is a physiological inducer of autophagy, and we used Hank's balanced salt solution (HBSS), a known autophagy stimulus, which caused robust degradation of IRF3 protein in a time-dependent manner (Fig. 5*A*). We tested whether HBSS treatment affects the phosphorylation and subsequently the transcriptional activity of IRF3. HBSS strongly inhibited the RLR-induced p-IRF3 (pSer^396^) (Fig. 5*B*). As a result, the RLR-induced expression of IFIT3 was inhibited by HBSS (Fig. 5*C*). To further strengthen these results, we analyzed the RLR-induced IFIT3 (Fig. 5*D*) and IFNβ (Fig. 5*E*) mRNA levels, which were inhibited by HBSS in MDA-MB-453 cells. Similarly, RLR-induced Ifit1 gene expression was also inhibited by HBSS in RAW264.7 cells (Fig. 5*F*). The activation and stability of TBK1, the kinase that phosphorylates IRF3, were relatively less affected by HBSS (Fig. 5*G*). The RLR-mediated activation of p38 MAPK, a stress-inducible kinase was also unaffected by HBSS (Fig. 5*H*). Since IRF3 is a major target of autophagic degradation, we tested whether HBSS-mediated suppression of IFIT3 gene induction can be alleviated by overexpression of IRF3. Indeed, in U4C cells, derived from HT1080 cells but cannot induce IFIT3 by IFN-signaling (28), the overexpression of IRF3 rescued the HBSS-mediated inhibition of IFIT3 gene induction by RLR (Fig. 5*I*).

**Figure 6:**
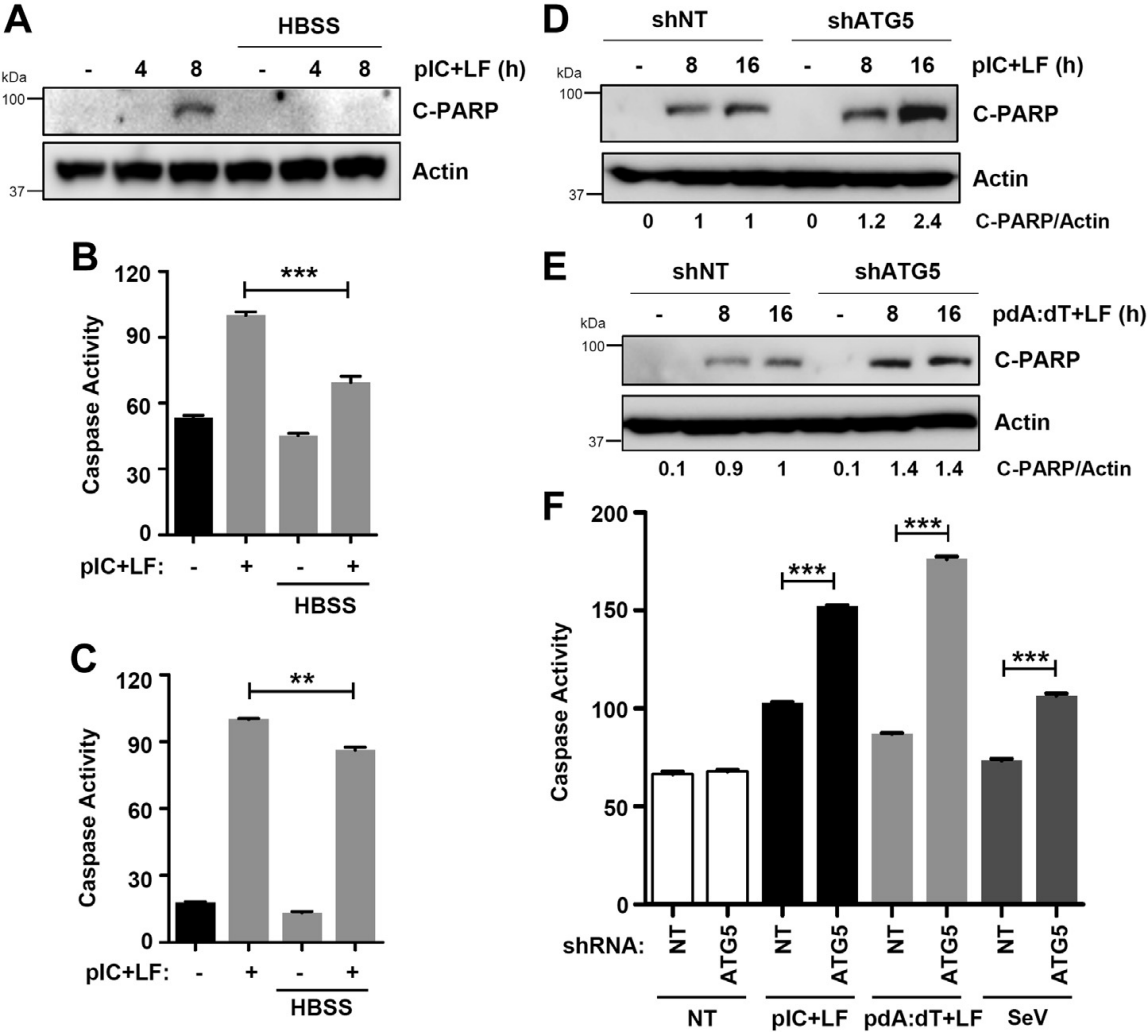
**Autophagic degradation inhibits the proapoptotic activity of IRF3.***A*, MDA-MB-453 cells were transfected with polyI:C (pIC+LF) in the absence or the presence of Hank's balanced salt solution (HBSS) for the indicated times when the levels of cleaved PARP were analyzed by immunoblot. *B* and *C*, MDA-MB-453 (*B*) or HT1080 (*C*) cells were transfected with polyI:C (pIC+LF) in the absence or the presence of HBSS, and the caspase-3 activity was measured. *D* and *E*, HT1080 cells, stably expressing nontargeting (NT) or ATG5-specific shRNA, were transfected with polyI:C (pIC+LF, *D*) or poly(dA:dT) (*E*) for the indicated times when the levels of cleaved PARP were analyzed by immunoblot. *F*, HT1080 cells, expressing nontargeting (NT) or ATG5-specific shRNA, were transfected with poly(I:C) (pIC+LF) or poly(dA:dT), or infected with Sendai virus (SeV) for 24 h, when the caspase-3 activity was measured. ∗ indicates *p* < 0.05.

IRF3 is activated differentially for the transcriptional and proapoptotic activities; IRF3 mutants can function pathway-specifically (5). Therefore, we examined whether autophagy activation can suppress the proapoptotic activity of IRF3. In HBSS-treated cells, the proapoptotic activity of IRF3, analyzed by C-PARP, was strongly inhibited (Fig. 6*A*), indicating that autophagy also inhibits the proapoptotic activity of IRF3. Similarly, HBSS treatment also inhibited the RLR-induced caspase-3 activity in MDA-MB-453 (Fig. 6*B*) and HT1080 (Fig. 6*C*) cells. In the next series of experiments, we examined genetically whether the proapoptotic activity is enhanced in autophagy-deficient cells. Cytosolic dsRNA (polyI:C)-stimulated RIPA, analyzed by C-PARP, was enhanced in ATG5-knockdown (shATG5) HT1080 cells (Fig. 6*D*). Cytosolic dsDNA-stimulation promoted RIPA activity in ATG5-knockdown cells (Fig. 6*E*). We further validated these results by analyzing caspase-3 activity induced by dsRNA (polyI:C), dsDNA (polydA:dT), as well as Sendai virus (SeV) infection, all of which can trigger RIPA (Fig. 6*F*). Together, our results demonstrate that activation of autophagy by nutrient deprivation can suppress, whereas the ablation of autophagy can promote the IRF3 activities.

### Auranofin inhibits fatty-acid-induced hepatocyte apoptosis by autophagic degradation of IRF3

**Figure 7:**
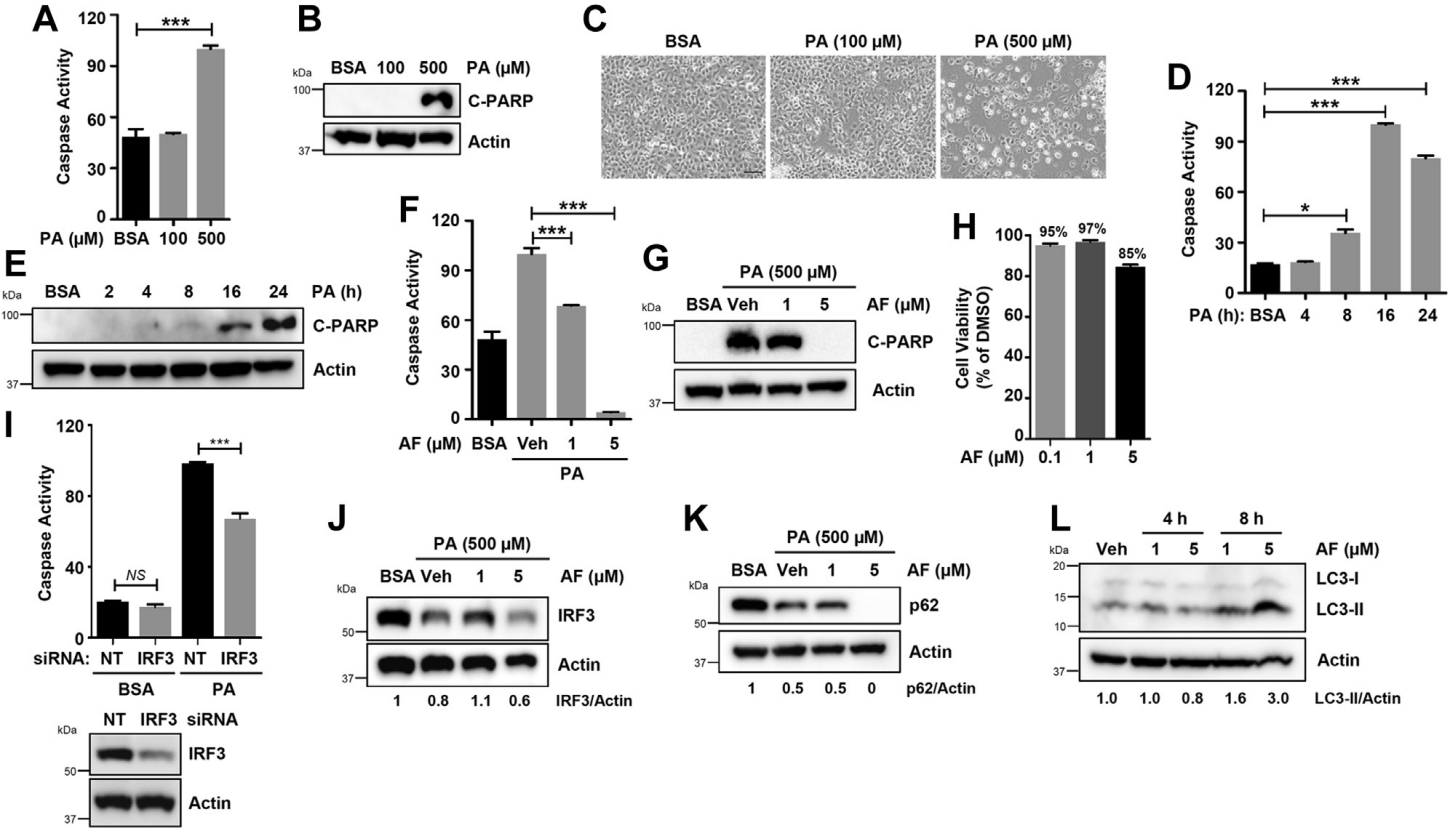
**Autophagic degradation of IRF3 by auranofin inhibits palmitic-acid-induced apoptotic cell death in human hepatocytes.***A*, Huh7 cells were treated with palmitic acid (PA) or vehicle (bovine serum albumin, BSA) for 24 h, when the caspase-3 activity was measured. *B* and *C*, Huh7 cells were treated with PA, and apoptotic cell death was analyzed by immunoblot of cleaved PARP (*B*) or bright field microscopy (*C*). *D* and *E*, Huh7 cells were treated with PA, and the caspase activity (*D*), and cleaved PARP (*E*) were analyzed at the indicated times post-treatment. *F* and *G*, Huh7 cells were pretreated with auranofin (AF) at the indicated concentrations and treated with PA. Caspase activity (*F*) and cleaved PARP (*G*) were measured 16 h posttreatment. *H*, Huh7 cells were treated with AF at the indicated concentrations for 8 h when cell viability was assessed by trypan blue exclusion assay. *I*, Huh7 cells were transfected with nontargeting (NT) or IRF3-specific siRNA, and the cells were analyzed for caspase-3 activity upon PA treatment for 16 h. The lower panel indicates the knockdown levels of IRF3 protein. *J* and *K*, Huh7 cells were pretreated with AF at the indicated concentrations and treated with PA. IRF3 (*J*) and p62 (*K*) were analyzed 16 h posttreatment. *L*, Huh7 cells were treated with AF, as indicated, and the LC3 levels were analyzed by immunoblot. Veh, Vehicle (DMSO), ∗ indicates *p* < 0.05, NS, nonsignificant, scale bar, 100 μm.

IRF3-mediated hepatocyte cell death contributes to alcoholic and nonalcoholic, liver diseases (11, 13). To examine whether auranofin can suppress hepatocyte apoptosis, we used the human hepatocyte cell line Huh7 and examined PA-induced apoptosis. PA treatment dose-dependently caused robust apoptosis, analyzed by caspase-3 activity (Fig. 7*A*) and C-PARP (Fig. 7*B*), compared with the vehicle (BSA) control. The PA-induced apoptosis also caused visible cell death in a dose-dependent manner (Fig. 7*C*). Next, we examined the kinetics of PA-induced apoptosis, and our results indicate that PA triggered caspase-3 activity (Fig. 7*D*) and C-PARP (Fig. 7*E*) in a time-dependent manner. Robust apoptotic activity was detected at 16–24 h post-PA treatment (Fig. 7, *D* and *E*). We used these optimal conditions (dose and time of PA treatment) to examine whether auranofin inhibits PA-induced hepatocyte apoptosis. PA-induced apoptosis, analyzed by caspase-3 activity (Fig. 7*F*) and C-PARP (Fig. 7*G*), was significantly inhibited by auranofin. The antiapoptotic activity of auranofin was not due to any cytotoxic effects (Fig. 7*H*). To investigate whether PA-induced apoptosis requires IRF3, we used the siRNA-mediated knockdown of IRF3 in Huh7 cells. The PA-induced apoptotic activity was significantly reduced in the IRF3-knockdown cells compared with the nontargeting control (Fig. 7*I*). Because IRF3 plays a proapoptotic role in hepatocyte apoptosis, we examined whether auranofin induces IRF3 degradation, as observed earlier in nonhepatic cells, to inhibit PA-induced apoptosis. PA treatment caused degradation of IRF3 protein, which was further enhanced by auranofin (Fig. 7*J*), suggesting that auranofin suppressed hepatocyte cell death by inhibiting IRF3's proapoptotic function. We examined whether auranofin activates autophagy, which is required for IRF3 degradation, in Huh7 cells. PA treatment caused degradation of the autophagic marker p62, and auranofin further enhanced this in a dose-dependent manner (Fig. 7*K*). Finally, we tested whether auranofin treatment, similar to other human and mouse cells, triggers autophagy in human hepatocytes. Our results indicate that auranofin treatment caused the accumulation of LC3-II, a marker of autophagosome formation, in Huh7 cells (Fig. 7*L*). Collectively, our results suggest that auranofin inhibits the fatty-acid-induced hepatocyte apoptosis by causing autophagic degradation of IRF3 protein.

## Discussion

**Figure 8:**
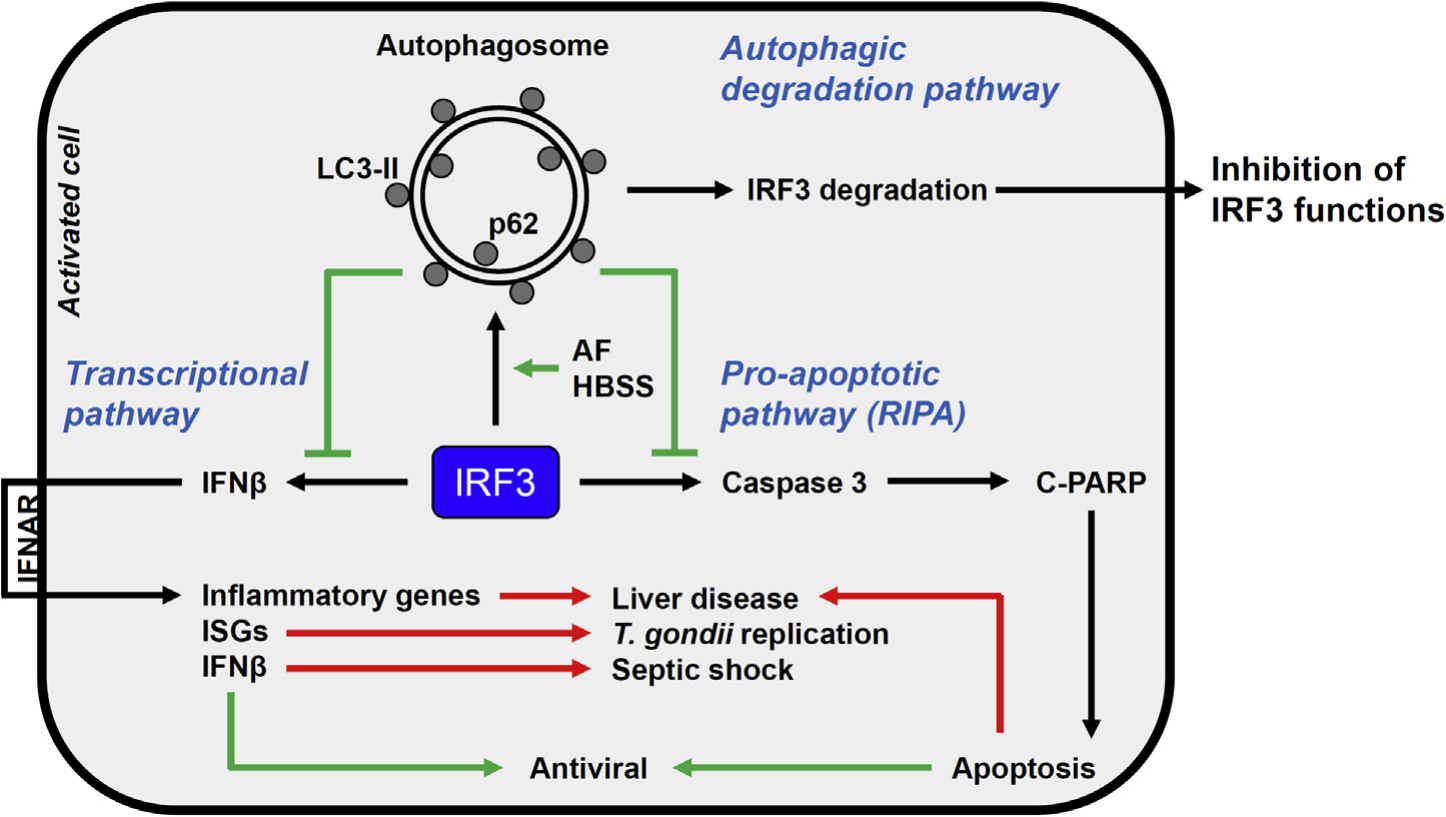
**Auranofin-induced autophagic degradation of IRF3 inhibits its cellular functions.** IRF3's transcriptional and RIPA activities contribute to the antiviral defense and can be harmful in inflammatory and metabolic diseases. A small-molecule, auranofin, promotes autophagic degradation of IRF3 protein, thus blocking both its transcriptional and RIPA functions.

Given the critical role of IRF3 in innate immune defense, identifying both positive and negative modulators of the IRF3 functions is desirable. Recently, we performed a high-throughput screen, which isolated a subset of FDA-approved drugs that promoted the proapoptotic activity of IRF3 (19). We showed that doxorubicin and pyrvinium pamoate inhibit virus replication by activating the IRF3 functions. In addition to activators, the screen isolated auranofin as an inhibitor of IRF3's proapoptotic pathway, RIPA. Our results demonstrate that auranofin inhibits both arms of IRF3's innate immune response. In this study, we demonstrated that auranofin induced cellular autophagy to degrade IRF3 protein; as a consequence, IFNβ production and IRF3-driven apoptosis were impaired. Genetic inhibition of autophagy restored IRF3 protein levels and concomitant activity. We evaluated the effect of auranofin in an *in vitro* cell culture model of liver disease, in which IRF3 plays a critical role (Fig. 8).

As the central component of antiviral immunity, careful regulation of IRF3 activity is crucial to facilitate a robust immune response to viral infection. However, IRF3 is also involved in the production of inflammatory cytokines, whose activity can be detrimental to the host when left unchecked. Therefore, the ability to quickly activate and inactivate IRF3 is paramount to maintaining immune homeostasis. One way the body ensures the return to basal levels of IRF3 activity is through the degradation of IRF3 protein, thus avoiding excessive inflammation. There are multiple routes by which IRF3 degradation may occur, and previously, we and others have reported a mechanism for apoptotic caspases to cleave IRF3, resulting in its proteasomal degradation (21, 29). IRF3 protein stability in virus-infected cells is also controlled through several members of the tripartite motif (TRIM) family of proteins. Virus infection induces the nuclear localization of TRIM26, which promotes K48-linked polyubiquitination of activated IRF3 to degrade IRF3 and thereby attenuate IFNβ production (30). Recently, TRIM21 was found to target IRF3 through precision autophagy (24, 25). Cross talk between autophagy and type-I IFN response was also explored in a study, which suggests that virus-induced polyubiquitinated IRF3 is targeted for autophagic degradation (26). It will be interesting to study whether auranofin modulates the TRIM-ubiquitin-autophagy mechanism to degrade IRF3. These results will have implications for the cellular functions of TRIM proteins as well. How auranofin triggers the autophagy pathway is not clear, and future studies will be required to address this question.

The role of autophagy during virus infection is inherently complex. Promotion of autophagy can be beneficial to the host by limiting destructive immune responses or harmful by eliminating IRF3-driven production of antiviral genes and apoptosis. We showed a proviral function of virus-induced autophagy in paramyxovirus replication, and an IFN-inducible protein inhibits this to suppress virus replication (27, 31). Viruses employ a number of strategies to subvert the innate immune system, including the targeting of IRF3. Since IRF3 is such a critical regulator of the antiviral response, it comes as no surprise that viruses degrade IRF3 to suppress its activity and thus prolong viral replication. Two nonstructural proteins encoded by respiratory syncytial virus (RSV), NS1 and NS2, mediate the formation of an NS-degradasome (NSD) complex to specifically target IRF3, among other innate immune factors, for removal from infected cells (32). In addition to producing viral enzymes (*e.g.*, proteases or ubiquitin ligases) capable of stimulating IRF3 degradation, viruses also hijack cellular machinery to degrade IRF3, resulting in the downregulation of IFN production and the inhibition of apoptosis. Thus, we speculate that cellular autophagy may represent yet another mechanism that some viruses can utilize to downregulate IRF3's antiviral function in innate immunity. Whether drug-induced IRF3 degradation favors virus replication will require an in-depth investigation.

In this study, we presented that the antirheumatic drug auranofin inhibited IRF3-mediated cell death in multiple cell lines and in response to the stimulation of several IRF3 activation pathways. Moreover, we found auranofin to strongly inhibit IRF3's function as a transcription factor, validating previously published results (33, 34). It has been reported *in vitro* that auranofin suppresses the release of IL-1β and TNFα from immune cells (35, 36). This suggests that auranofin acts at multiple points to shut down innate immune signaling and limit inflammatory cytokine production. Thus, the possible clinical use of auranofin extends beyond its original intended purpose for rheumatoid arthritis. Interestingly, a recent study revealed auranofin was antiviral against SARS-CoV-2 and attenuated inflammation that contributed to lung pathology, suggesting its application as an antiviral compound for respiratory viruses (37). Additionally, its role in suppressing inflammation points to a potential use against a range of autoimmune diseases, particularly those driven by IRF3 activity. Since IRF3-mediated apoptosis is also detrimental in liver diseases, we investigated the effect of auranofin on fatty-acid-induced cell death. This study and others indicate that autophagy induction may be a protective mechanism in liver disease (38, 39). It should be noted that the small molecules often have nonspecific effects and multiple cellular targets. Therefore, genetic studies are required to complement the results obtained from the chemicals for investigating the mechanisms of action. Overall, our data suggest that the cellular autophagy pathway modulates IRF3 function to suppress the innate immune response, which may be relevant in a range of illnesses.

## Experimental procedures

### Cells and reagents

The human cell lines HT1080 (ATCC CCL-121), MDA-MB-453 (ATCC HTB-131), Huh7, and U4C, and the mouse cell line RAW264.7 (ATCC TIB-71) were maintained in DMEM containing 10% FBS and antibiotics—penicillin and streptomycin. All cell lines used in the study were maintained in the authors' laboratory. Ligands for RIG-I and TLR3 have been described previously (5, 6, 40). Poly(dA:dT) and auranofin (#A6733) were obtained from Sigma-Aldrich. SeV Cantell strain was obtained from Charles River Laboratories, and the infection procedures have been previously described (3, 6). GFP-expressing VSV (gVSV) infection has been described previously (3). The antibodies against specific proteins were obtained as indicated: anti-cleaved PARP (Cell Signaling Technology #9546), anti-IFIT1 (described previously (5, 6)), anti-IFIT3 (described previously (5, 6)), anti-Actin (Sigma-Aldrich #A5441), anti-phospho-IRF3 (Ser^396^) (Cell Signaling Technology #4947), anti-Irf3 (Novus Biologicals # NBP1-78769 and described previously (5, 6)), anti-LC3 (Cell Signaling Technology #2775), anti-p62 (Fitzgerald #20R-PP001), anti-ATG5 (Cell Signaling Technology #2630), anti-BAX (Cell Signaling Technology #2772), anti-pTBK1 (Cell Signaling Technology #5483S), anti-TBK1 (Cell Signaling Technology #3013S), anti-RIG-I (Cell Signaling Technology #3743S), anti-MAVS (Santa Cruz Biotechnology #sc166583), anti-TRAF2 (Cell Signaling Technology #4724S), anti-pSTAT1 (Cell Signaling Technology #8826T), anti-STAT1 (Cell Signaling Technology #14994T), anti-p-p38 (Cell Signaling Technology #4511S), anti-p38 (Cell Signaling Technology #9212S), goat anti-guinea pig IgG (Novus Biologicals #NB7396), goat anti-mouse IgG (Rockland Biologicals #610-1319), and goat anti-rabbit IgG (Rockland Biologicals #611-1322).

### Gene knockdown and knockout

ATG5-specific shRNA (Sigma #SHCLNG-NM_004849) or nontargeting control shRNA was stably expressed by lentivirus, and transduced cells were selected in puromycin-containing medium, as previously described (27, 31). The Huh7 cells were transfected with either IRF3-specific (L-006875-00-0005) or non-targeting (D-001810-10-20) siRNAs, obtained from Horizon Discovery Bioscience Limited, using Dharmafect 4 reagent (T-2004-03, Horizon Discovery Bioscience Limited) and used for the palmitic acid-mediated apoptosis assay. The IRF3^*−*/*−*^ HT1080 cells were generated using CRISPR/Cas9 technology, as described previously (19).

### Transfection and treatment

RLR stimulation, indicated by pIC+LF in the figures and legends, was performed by transfecting cells with polyI:C (pIC) using Lipofectamine 2000 (Thermo Fisher Scientific) for 8–24 h. Similarly, cells were transfected with poly(dA:dT) using Lipofectamine 2000 (LF) according to the manufacturer's instructions. TLR3 stimulation was achieved by directly adding polyI:C (25 μg/ml) to the media for 8 h. Cells were then analyzed for caspase activity, protein levels *via* immunoblot, or mRNA levels by qRT-PCR, as indicated in the figure legends. For drug treatments, cells were pretreated with auranofin (5 μM, unless otherwise specified in the figure legends) for 1 h prior to poly(I:C) or poly(dA:dT) transfection or PA treatment. DMSO was used as vehicle control for auranofin. For PA treatment, PA was prepared in BSA-containing DMEM, as described before (15), at the final indicated concentrations and added directly to the culture media for the indicated length of treatment.

### Cell lysis and immunoblot

Immunoblot analyses were performed using previously described procedures (5, 6). Cells were lysed in 50 mM Tris buffer, pH 7.4 containing 150 mM of NaCl, 0.1% Triton X-100, 1 mM sodium orthovanadate, 10 mM sodium fluoride, 10 mM β-glycerophosphate, 5 mM sodium pyrophosphate, protease and phosphatase inhibitors (Roche). Total protein extracts were analyzed by SDS-PAGE followed by immunoblot. Immunoblots were quantified using ImageJ software and normalized to Actin, as indicated in each figure.

### Caspase activity assay

The caspase-3/7 activity of the cell lysates was analyzed using previously described procedures (5, 6). Cell lysates were used to measure caspase activity using the Apo-ONETM Homogenous Caspase-3/7 Assay according to the manufacturer's instructions (Promega). Caspase activity was normalized to protein concentration, and RLR-stimulated or PA-stimulated cells were arbitrarily set at 100, and all the other values were normalized to this (as in Fig. 1*C*).

### Light microscopy images

Cells were seeded in a 6-well plate and were treated the following day as indicated in the figure legends. Prior to imaging, cells were washed with PBS and replaced with fresh media. Culture fields were imaged 8–24 h post-treatment, and images presented here are representative results.

### RNA isolation and qRT-PCR analyses

Total RNA was isolated using Trizol (Invitrogen) 8 h post-treatment, cDNA was prepared using ImProm-II Reverse Transcription Kit (Promega), and the cDNA was analyzed using RadientTM SYBR Green PCR mix (alkali Scientific Inc.) in Roche LightCycler 96 instrument and analyzed with the LightCycler 480 Software, Version 1.5. The expression levels of the mRNAs were normalized to 18S rRNA. For the qRT-PCR analyses of the respective genes, the following primers were used:

IFIT1-fwd: TCTCAGAGGAGCCTGGCTAAG
IFIT1-rev: GTCACCAGACTCCTCACATTTGC
IFIT3-fwd: GAACATGCTGACCAAGCAGA
IFIT3-rev: CAGTTGTGTCCACCCTTCCT
IFNB1-fwd: CGCCGCATTGACCATCTA
IFNB1-rev: GACATTAGCCAGGAGGTTCT
Ifit1-fwd: CAGAAGCACACATTGAAGAA
Ifit1-rev: TGTAAGTAGCCAGAGGAAGG
18S-fwd: ATTGACGGAAGGGCACCACCAG
18S-rev: CAAATCGCTCCACCAACTAAGAACG

### Cell viability assay

A trypan blue exclusion assay was performed to determine the cell viability of auranofin-treated cells. HT1080 or Huh7 cells were seeded in duplicate in a 12-well plate. The following day, cells were treated with the indicated concentrations of auranofin or DMSO as the vehicle control. The treated cells were trypsinized, resuspended in complete DMEM, and stained with trypan blue 8 h posttreatment. Live and dead cells were counted using a hemocytometer, and cell viability was calculated relative to the DMSO control.

### Statistical analyses and software

The results presented here are the representatives of at least three independent experiments. The statistical analyses were performed using GraphPad Prism 5.02 software. The values presented in the graphs indicate the mean ± SEM, collected from both technical and biological replicates. The *p* values were calculated using two-tailed, independent student's *t* test. A *p* value <0.05 indicates statistical significance.

## Data availability

All data presented in this paper are contained within the manuscript.

## Conflict of interest

The authors declare that they have no conflicts of interest with the contents of this article.
